# Understanding key drivers of job satisfaction in primary health care organizations in Kazakhstan: a Herzberg's two-factor theory-based qualitative study

**DOI:** 10.3389/frhs.2026.1900594

**Published:** 2026-08-11

**Authors:** Ainara Oraltayeva, Claire Chaumont, Lyazzat Kosherbayeva, Kuanysh A. Yergaliyev

**Affiliations:** 1 School of Medicine, Nazarbayev University, Astana, Kazakhstan; 2 Harvard T.H. Chan School of Public Health, Boston, MA, United States; 3 Department of Health Policy and Management, Asfendiyarov Kazakh National Medical University, Almaty, Kazakhstan

**Keywords:** health policy, health workforce, Herzberg's two-factor theory, job satisfaction, Kazakhstan, primary health care, qualitative research

## Abstract

**Background:**

Primary healthcare (PHC) is the first point of contact between populations and health systems, and physician workforce satisfaction is central to its effective functioning. In Kazakhstan, despite successive healthcare reforms, public polyclinics face persistent workforce challenges including high burnout rates, turnover, and low morale. Empirical evidence specifically examining the drivers of job satisfaction among physicians in public primary care organization remains limited. This study explored motivational and hygiene factors influencing job satisfaction and dissatisfaction among physicians working in public polyclinics in Astana, Kazakhstan, guided by Herzberg's Two-Factor Theory.

**Methods:**

A qualitative study using in-depth semi-structured interviews was conducted between January and March 2025. Seventeen physicians from three public polyclinics in Astana were recruited through purposive and snowball sampling. Interview guides followed Herzberg's motivator and hygiene factor domains. Data were analyzed thematically, with the theory serving as the organizing structure. Trustworthiness was ensured through peer debriefing, field notes, reflexivity and thick description.

**Results:**

Nine themes emerged across two categories. Hygiene factors generating dissatisfaction included inadequate salary and limited non-monetary benefits, poor infrastructure, hierarchical management and limited autonomy, and job instability linked to mandatory work obligations. Motivator factors sustaining engagement included intrinsic satisfaction from patient care, supportive colleagues, commitment to professional excellence, continuing medical education and recognition. Salary was the most dominant source of dissatisfaction. Participants indicated that compensation issues needed to be resolved first before any social intervention strategies could be meaningfully assessed.

**Conclusions:**

Physicians in Kazakhstani public primary healthcare organizations are sustained primarily by intrinsic motivators despite pervasive extrinsic dissatisfiers. Findings map onto Herzberg's Two-Factor Theory and suggest priorities for PHC human resources for health (HRH) policy in Kazakhstan, including compensation reform, workload rationalization, reduced administrative burden, and investment in non-monetary recognition. These findings provide an evidence base for HRH reform within Kazakhstan's ongoing Primary Health Care transformation agenda.

## Introduction

Job satisfaction among healthcare professionals is a critical determinant of workforce retention, quality of care, and organizational performance (1, 2). In primary healthcare (PHC) settings, where professionals face high patient volumes, emotional labor, and administrative pressure, maintaining satisfaction is particularly challenging (3). Low job satisfaction is consistently associated with burnout, absenteeism, turnover intention, and diminished quality of care (4–6), while satisfied professionals demonstrate greater motivation and commitment to patient-centered practice (7). In fact, according to the Job Demands-Resources theory, individual job characteristics, such as workload, organizational support, autonomy or development opportunities can all impact burnout risk (8).

PHC systems serve as the cornerstone of equitable and sustainable health service delivery, providing preventive services, early detection, and continuity of care (9). In Kazakhstan, public polyclinics are the primary entry point to care for the majority of the population. These multidisciplinary organizations provide general, specialized, and preventive services under a capitation payment model with pay-for-performance component (10). Despite this structure, service tariffs and physician salaries remain comparatively low, a circumstance that may contribute to professional dissatisfaction and burnout of PHC professionals (10–12).

Kazakhstan has implemented successive healthcare reforms since its independence from the Soviet Union in 1991, aiming to modernize the PHC sector and improve system efficiency. Nevertheless, its healthcare professionals continue to face significant challenges. The Organization for Economic Co-operation and Development (OECD) health system review identified persistent challenges in equity, access, and workforce efficiency (11). National data indicate that approximately 31% of physicians report burnout symptoms, and 3.7% show signs of severe depression (13). Overall morale, encompassing motivation, job satisfaction, and psychological well-being, has been reported as low among PHC providers (10, 13). Recent national analyses highlight persistent human resources for health (HRH) challenges in Kazakhstan, including a low share of generalist practitioners in PHC (11.6% in 2022) and continued rural–urban maldistribution of the health workforce (14).

The literature identifies multiple determinants of healthcare worker job satisfaction including work environment, clinical autonomy, compensation, workload, professional development opportunities, and interpersonal relationships (15–18). Workload imbalance and work–life conflict are additional contributors to burnout, particularly when combined with limited organizational support (8, 19). On the other hand, interpersonal relationships and effective teamwork are positively associated with job satisfaction and retention (20, 21).

Herzberg's Two-Factor Theory (originally published in 1959 by Herzberg, Mausner, and Snyderman) provides a well-established framework for disentangling sources of satisfaction from sources of dissatisfaction (22). The theory distinguishes motivator factors—intrinsic to the work itself, including achievement, recognition, responsibility, and professional growth from hygiene factors extrinsic conditions such as salary, interpersonal relations, supervision, and working conditions. Crucially, hygiene factors prevent dissatisfaction when adequate, but do not generate positive satisfaction; only motivators generate active engagement. Although the theory has been critiqued on methodological grounds and for its cross-cultural assumptions (23), it has been widely applied and validated in healthcare workforce research, particularly across low- and middle-income country (LMIC) settings (24–26).

Despite a growing body of global evidence, qualitative research specifically examining physician job satisfaction in public polyclinic settings in Kazakhstan remains limited. Most existing studies address general workforce issues or focus on nursing rather than the unique organizational context of PHC organizations (13). Given the strategic importance of PHC in Kazakhstan's Universal Health Coverage agenda and persistent retention concerns in this sector, deeper contextual understanding of satisfaction drivers is required to inform actionable HRH policy.

Guided by Herzberg's Two-Factor Theory, this study aimed to explore the key drivers of job satisfaction and dissatisfaction among physicians working in public polyclinics in Astana, Kazakhstan's capital city, and to examine the role of social intervention strategies in addressing workforce dissatisfaction.

## Materials and methods

### Study design and setting

A qualitative study design using in-depth semi-structured interviews was employed to explore physicians' lived experiences of job satisfaction and dissatisfaction. Qualitative methodology was selected to capture the nuanced, context-dependent factors that quantitative measures are unable to fully capture (27). The study was conducted in public polyclinics in Astana, the administrative capital of Kazakhstan, selected due to the city's concentration of PHC facilities, its diverse patient population, and its centrality to national health reform initiatives. Three public polyclinics were purposively selected to capture organizational diversity.

### Data collection

In-depth semi-structured interviews were conducted between January and March 2025. Interview guides were developed based on Herzberg's Two-Factor Theory domains: motivator factors (achievement, recognition, the work itself, responsibility, advancement) and hygiene factors (salary, interpersonal relations, supervision, working conditions, company policy). Interviews lasted 40–60 min and were conducted face-to-face or via video online platform (Teams/Zoom), according to participant preference. All interviews were audio-recorded with written informed consent and subsequently transcribed verbatim. Transcripts were reviewed against the original recordings to ensure accuracy. Minor disfluencies that did not affect meaning like filler words were retained where appropriate, while non-verbal behaviors, overlapping speech, laugher, sighs, etc. and detailed conversational features were not systematically documented because they were not relevant to the study's interpretive aims and the study focused on the content and meaning of participants' accounts rather than conversation analysis. The transcripts were subsequently verified against the original audio recordings by a second member of the research team to ensure transcription accuracy and completeness. Any discrepancies were resolved through discussion and consensus. Prior to analysis, all transcripts were de-identified by removing or replacing personal and contextual identifiers, including participants' names, their coworkers' names, the names of primary healthcare facilities, geographic locations, and any other information that could reasonably identify an individual or organization. Each participant was assigned a unique study identification code.

### Conceptual framework

Herzberg's Two-Factor Theory (22) distinguishes between factors that generate positive job satisfaction (motivators) and factors that, if absent or inadequate, cause dissatisfaction (hygiene factors). This distinction is theoretically important: improving hygiene factors reduces dissatisfaction but does not create satisfaction; only motivators generate active engagement and high performance. Table 1 summarizes the framework as applied in this study.

**Table 1:** Application of Herzberg's two-factor theory to the study.

Motivator factors (generate satisfaction)	Hygiene factors (prevent dissatisfaction)
Achievement/Intrinsic satisfaction from patient care	Salary and compensation
Recognition from patients and peers	Interpersonal relations (colleagues, management)
The work itself (meaning, purpose)	Working conditions and infrastructure
Professional growth and continuing education	Supervisory practices and autonomy
Responsibility and clinical autonomy	Organizational policy and job stability

Adapted from Herzberg et al. (22); Alshmemri et al. (25).

### Data analysis

Data were analyzed using thematic analysis with a deductive approach where an established theoretical framework guides the inquiry (28). The analysis followed five stages: familiarization with transcripts; development of a theoretically-derived coding framework based on Herzberg's domains from 20% of transcripts; applying codes to remaining transcript material; organizing codes by theme; and interpretation. Coded data were managed using NVivo 14 software. An initial codebook was developed, iteratively refined, and reviewed by the researchers through peer debriefing sessions. Conflicting interpretations were resolved through discussion until consensus was reached. The interview guide (Supplementary Table S1) and the qualitative codebook (Supplementary Table S2) used for thematic analysis are provided in the Supplementary Material.

### Trustworthiness

Trustworthiness was addressed following Lincoln and Guba's criteria (29): credibility was supported by peer debriefing, use of direct participant quotations, and maintenance of field notes documenting contextual observations during interviews; transferability by provision of thick contextual descriptions; and dependability by documenting the analytic process and revisiting coding multiple times. Reflexivity was maintained throughout the study through reflective notes in which the researchers critically examined their assumptions, positionality, and potential influence on data interpretation, thereby strengthening analytic transparency and trustworthiness. Written informed consent was obtained from all participants prior to interviews. In addition, reporting of the study followed the Consolidated Criteria for Reporting Qualitative Research (COREQ): 32-item checklist, with the completed checklist provided in Supplementary Table S3.

## Results

### Participants and sampling

The target population comprised licensed medical doctors employed in public polyclinics for a minimum of six months. This criterion ensured participants could provide substantive reflections on the working environment. Medical students, interns, and residents were excluded, but state-scholarship recipients currently fulfilling their mandatory post-graduate posting were included.

Purposive sampling was used initially to recruit participants representing diverse specializations and experience levels, supplemented by snowball sampling. Participants were recruited via direct outreach, institutional flyers, and social media. Interviews continued until thematic saturation was reached at 17 participants.

Seventeen physicians participated across three polyclinics in Astana: 14 general practitioners, one pediatrician, one surgeon, and one oncologist. The sample was 88% female, reflecting the gender distribution characteristic of PHC physician workforces in Kazakhstan. Table 2 presents participant characteristics.

**Table 2:** Characteristics of study participants (*n* = 17).

Characteristic	*n*	%
Polyclinic 1	7	41
General Practitioner	6	35
Pediatrician	1	6
Polyclinic 2	6	35
General Practitioner	5	29
Surgeon	1	6
Polyclinic 3	4	24
General Practitioner	3	18
Oncologist	1	6
Across all three polyclinics		
Gender: Female	15	88
Gender: Male	2	12
Experience ≥6 months (inclusion criterion met)	17	100

The analysis identified nine themes organized within Herzberg's two-factor framework: four hygiene factors and five motivator factors. Table 3 presents the thematic structure.

**Table 3:** Thematic structure of findings mapped to Herzberg's Two-factor theory.

Factor type	Theme	Core issue
Hygiene factors	1. Inadequate salary and absence of non-monetary benefits	Low pay relative to workload; lack of social welfare benefits
	2. Poor workplace infrastructure	Outdated equipment, unreliable internet, unsuitable facilities
	3. Hierarchical management and limited clinical autonomy	Protocol rigidity; centralized decision-making; disempowerment
	4. Conditional job stability (mandatory work obligation)	Retention driven by obligation, not motivation
Motivator factors	5. Intrinsic satisfaction from patient care	Passion for the profession; meaning derived from patient recovery
	6. Supportive collegial relationships	Teamwork; mutual assistance; positive work atmosphere
	7. Commitment to professional excellence	Preference for clinical expertise over administrative advancement
	8. Continuing medical education (CME)	Regular re-certification; conference participation; skill development
	9. Diminished societal recognition of the physician role	Perceived loss of professional status; patient skepticism

### Hygiene factors

#### Theme 1: inadequate salary and absence of non-monetary benefits

Inadequate salary dissatisfaction was the most pervasive and consistently articulated grievance across all 17 participants. Physicians described their compensation as disproportionate to workload, professional responsibility, and societal risk. The pay-for-performance (PFP) approach of remuneration was acknowledged as a positive mechanism but insufficient to substitute for baseline salary increases.

“The salary does not match the workload. We are responsible for people's lives and earn less than a salesperson in a supermarket.” [P4, General Practitioner]

“The Performance-based payment (PBP) is a nice bonus, but if we are going to improve something, it would be better to raise wages.” [P4, General Practitioner]

Beyond salary, participants described an absence of effective non-monetary benefits. Social welfare provisions notionally available to healthcare workers—including priority housing loans and subsidized childcare placement—were described as inaccessible or nominal in practice.

“They say there are programs for teachers and doctors, but in practice there is no real help.” [P6, General Practitioner]

#### Theme 2: poor workplace infrastructure

Participants consistently reported inadequate physical working conditions, including outdated equipment, unreliable internet connectivity, and deteriorating facility infrastructure. These deficiencies were experienced as direct impediments to clinical effectiveness and patient care quality.

“The clinic is located in an old building. There are no windows in offices, high humidity, no access to fresh air—it is especially difficult in summer due to heat and lack of air conditioning.” [P3, General Practitioner]

“Sometimes the equipment is outdated, making our job harder.” [P5, Surgeon]

#### Theme 3: hierarchical management and limited clinical autonomy

Participants described a management culture characterized by top-down directives, protocol rigidity, and limited scope for physician input into clinical or organizational decisions. While formal communication channels (group meetings, messaging platforms) existed, many participants expressed skepticism about their effectiveness in generating change.

“Key decisions are made by leadership. There is not much room for suggestions from doctors.” [P8, General Practitioner]

“We have group chats and meetings, but they do not always lead to real changes.” [P3, General Practitioner]

Some participants reported experiencing or witnessing public humiliation of clinical staff by senior administrators, which further eroded trust and professional dignity.

#### Theme 4: conditional Job stability (mandatory work obligation)

A substantial proportion of participants remained in public polyclinic employment primarily to fulfill mandatory post-graduation work obligations tied to state scholarships, rather than out of voluntary commitment. Physicians described this as a source of psychological disengagement, with those approaching the end of their obligation period expressing clear intentions to transition to private sector employment.

“Many doctors stay just to fulfil their required service years. There is little actual motivation to remain long-term.” [P9, General Practitioner]

### Motivator factors

#### Theme 5: intrinsic satisfaction from patient care

Despite pervasive dissatisfaction with extrinsic conditions, all 17 participants identified intrinsic satisfaction from patient care as a primary source of professional fulfilment. The emotional rewards of positive clinical outcomes, direct patient interaction, and patient gratitude were described as the dominant drivers sustaining continued commitment.

“Only love for my work and love for my patients motivates me.” [P2, General Practitioner]

“Working with children is a very special atmosphere. This largely compensates for the numerous disadvantages of my profession.” [P1, Pediatrician]

“The most satisfying thing is direct work with patients—communication, helping them, treatment, diagnostics.” [P3, General Practitioner]

#### Theme 6: supportive collegial relationships

Peer support was identified by all participants as a significant enabler of day-to-day wellbeing. Collegial solidarity—particularly during emergencies, emotionally demanding cases, and periods of high workload—was described as a protective factor against burnout and a meaningful source of professional belonging.

“We have a warm and friendly atmosphere; everyone helps each other.” [P3, General Practitioner]

“I enjoy working here because my colleagues are supportive.” [P5, General Practitioner]

#### Theme 7: commitment to professional excellence

Participants articulated a strong preference for deepening clinical expertise rather than pursuing administrative career pathways. Professional identity was closely tied to competence in clinical practice and quality of patient care, rather than hierarchical advancement.

“I strive for professional excellence in my medical practice rather than career advancement in administration.” [P5, Surgeon]

#### Theme 8: continuing medical education (CME)

Participation in CME activities—including re-certification programs, specialist courses, and professional conferences—was viewed positively as a mechanism for skill development and professional identity reinforcement. Although mandatory re-certification every five years was acknowledged as a regulatory requirement, many participants reported voluntarily pursuing additional training.

“Medicine constantly changes, protocols and treatment approaches get updated, so we have to keep learning.” [P1, Pediatrician]

#### Theme 9: diminished societal recognition of the physician role

Many participants described a perceived erosion of the social status of the medical profession, including patient skepticism, perceptions of physicians as service personnel rather than specialists, and the absence of formal public recognition mechanisms. Participants described the absence of professional recognition as diminishing motivation and professional fulfilment with is consistent with Herzberg's Two-Factor Theory, while also linking it to broader organizational and societal contexts that contributed to negative work experiences.

“The role of a doctor is somewhat diminished. We are perceived more as service personnel than as specialists.” [P1, Pediatrician]

### The role of social interventions

When asked about existing social intervention strategies (wellness programs, team-building activities), most participants were either unaware of available provisions or reported that nominal programs did not translate into accessible, meaningful support. Critically, participants indicated that the effectiveness of any social intervention is contingent on prior resolution of the salary issue, which dominated the psychological space of professional dissatisfaction.

“There are no health or stress management programs. We just cope on our own.” [P10, Oncologist]

## Discussion

This study provides a theoretically grounded, empirically rich account of job satisfaction drivers among physicians in Kazakhstani public primary healthcare organizations (polyclinics). Applying Herzberg's Two-Factor Theory as a deductive framework, findings reveal a consistent pattern: physicians are sustained by powerful intrinsic motivators—principally the meaning derived from patient care and the solidarity of collegial relationships while simultaneously burdened by a constellation of hygiene deficits that generate chronic dissatisfaction.

The primacy of intrinsic satisfaction from patient care as a protective motivator aligns with findings from LMIC healthcare workforce studies across sub-Saharan Africa, South Asia, and Southeast Asia (24, 25), as well as with evidence from qualitative physician studies in the Central Asian context (13). This suggests that vocational commitment to medicine functions as a cross-contextual resilience resource. However, as Bonenberger et al. (26) demonstrated in Ghana, intrinsic motivation alone is insufficient to sustain retention when extrinsic conditions like salary fall below a critical threshold which is a dynamic, we clearly observe in our study.

In the study, salary dissatisfaction emerged as the dominant hygiene deficit, consistent with evidence from Kazakhstan-specific analyses (10, 13) and corroborated by international evidence demonstrating that perceived pay inequity relative to workload is a leading predictor of turnover intention (30). The performance-based payment system operating in Kazakhstani polyclinics, while conceptually aligned with value-based incentive structures was perceived by participants as an inadequate substitute for baseline salary reform. Nevertheless, salary reform should be considered within the broader context of public sector remuneration, where across-the-board salary increases have important fiscal and equity implications. These findings suggest that strengthening existing incentive mechanisms through greater transparency, fairness, and meaningful performance metrics, while ensuring adequate baseline remuneration, may represent a more feasible and sustainable policy approach.

The finding that collegial relationships constitute a primary motivator extends evidence from large-scale organizational health studies demonstrating that peer support mitigates burnout and enhances retention (20, 21). However, the sharp contrast between positive collegial relations and negative management relationships with the latter characterized by hierarchical control, protocol rigidity, and limited clinical autonomy was particularly observed. Restricted physician autonomy is a well-established predictor of turnover intention in primary care internationally (18), and the polyclinic system's centralized, protocol-driven management model appears to systematically undermine physicians' sense of professional agency in our study.

The mandatory post-graduation work obligation represents a structural feature of Kazakhstan's medical education financing system that warrants specific policy attention. Participants' descriptions of remaining in the polyclinic primarily to fulfil obligations, followed by departure to the private sector upon completion indicates a systemic retention failure that social interventions alone cannot address. Similar patterns of state-scholarship-driven retention instability have been documented in other post-Soviet health systems (13) and represent a structural HRH challenge requiring longitudinal policy solutions.

The inability of participants to meaningfully evaluate social intervention strategies due to overriding salary concerns underscores a theoretically consistent insight: hygiene deficits must be addressed to a minimally sufficient level before motivator-enhancing interventions can generate meaningful effects (22). This sequencing logic, embedded in Herzberg's original formulation, has direct programmatic implications for Kazakhstan's HRH reform agenda.

The predominance of women in this sample (88%) reflects the feminization of PHC physician workforces common across post-Soviet health systems. This gender distribution is broadly consistent with previous reports describing the predominantly female physician and health workforce in Kazakhstan and other post-Soviet countries (31). While the sample was not intended to be statistically representative, future research should explicitly examine whether gender intersects with job satisfaction in this context, particularly given documented gender gaps in salary equity and career advancement opportunities.

Those findings are not directly transferable to other regions of Kazakhstan, considering regional polyclinics outside of Astana may differ in staffing levels, infrastructure, patient volume, and access to professional development. Nevertheless, they provide helpful context to Kazakhstan's PHC transformation agenda.

### Limitations

This study has several limitations. First, the sample was relatively small and drawn exclusively from public polyclinics in Astana, a large urban capital, limiting transferability to rural, peri-urban, or regional health system contexts where working conditions and physician profiles may differ substantially. Second, the study focused solely on physicians; the perspectives of nurses, midwives, and allied health professionals, who constitute the majority of PHC staff, were not captured. Third, the mandatory work obligation context means the sample may over-represent physicians with lower voluntary commitment to the sector, potentially introducing sampling bias, although this overrepresentation is a finding in itself. Fourth, participant responses may have been shaped by social desirability bias, due to cultural norms that emphasize politeness, respect, and the avoidance of offending others. Finally, our study uses a predominantly deductive framework analysis guided by Herzberg's Two-Factor Theory. While this approach provided a structured basis for interpretation, it may have limited the identification of themes beyond the predefined theoretical framework. Future studies could adopt a mixed deductive–inductive approach to capture additional context-specific insights.

## Conclusions

This qualitative study provides evidence that physicians in Kazakhstani public polyclinics are primarily sustained by intrinsic motivation, specifically the meaning of patient care and collegial solidarity while experiencing chronic dissatisfaction driven by inadequate compensation, poor infrastructure, limited autonomy, and institutional instability. These findings are consistent with and extend Herzberg's Two-Factor Theory in a Central Asian PHC context. By providing context-specific qualitative evidence from an underrepresented healthcare setting, this study contributes to the broader discourse on health workforce motivation by demonstrating how established motivational theories operate within the unique organizational and policy context of Kazakhstan's primary healthcare system.

The study has direct implications for Kazakhstan's PHC transformation agenda and broader HRH policy. Priority actions should include: (1) a substantive revision of polyclinic physician salary structures, with particular attention to base compensation equity relative to workload; (2) rationalization of administrative and documentation burden to reclaim clinical time; (3) improvement of physical working conditions, including infrastructure, equipment, and digital connectivity; (4) reform of management practices to support participatory decision-making and physician autonomy; (5) development of accessible, meaningful non-monetary benefit programs; (6) longitudinal policy solutions to address retention beyond the mandatory obligation period; and (7) public recognition campaigns to restore the social status of the physician profession.

Future research should employ mixed methods to quantify the relative weight of satisfaction drivers identified here, extend inquiry to rural and regional polyclinic settings, examine the experiences of non-physician PHC cadres and assess the impact of gender on job satisfaction. Evaluations of targeted HRH interventions, particularly salary reform, optimization of performance-based incentive systems, and management development programs, are urgently needed to generate actionable evidence for policy.

## Data Availability

The original contributions presented in the study are included in the article/Supplementary Material, further inquiries can be directed to the corresponding author.
