# Supplementary material for: Understanding key drivers of job satisfaction in primary health care organizations in Kazakhstan: a Herzberg's two-factor theory-based qualitative study
Source: Front Health Serv. 2026 Aug 11;6:1900594. doi: 10.3389/frhs.2026.1900594 (PMC13503204; doi:10.3389/frhs.2026.1900594)
Supplement: Supplementary file 1 [file Table1.docx]

**Table S1. The Interview guideline**

| **Category** | **Questions** |
| --- | --- |
| **Hygiene Factors** |  |
| Salary | Do you consider your salary fair? Does it affect your job satisfaction?  What types of compensation or benefits do you receive? |
| Working conditions | What conditions has your organization created to ensure job stability? What factors contribute to this?  How would you describe the work atmosphere in your organization?  What aspects of your work environment help you stay productive? |
| Company policy | What rules and policies exist in your organization?  How would you evaluate the corporate culture in your organization? |
| Supervision | How would you assess the current communication with your management? |
| Interpersonal relationships | How would you describe the work atmosphere in your organization?  Are there any of-work activities organized? If so, what are they? Do you find them important? |
| **Motivating Factors** |  |
| Meaning of work | Which aspects of your job bring you the most satisfaction? |
| Responsibility and initiative | What are your career growth prospects? How does this affect your motivation?  Have you participated in any training programs or courses to enhance your qualifications? |
| Career growth | What are your career growth prospects? How does this affect your motivation?  Have you participated in any training programs or courses to enhance your qualifications? |
| Recognition and achievements | Do you receive enough recognition for your work? How does it motivate you? |
| Work itself | What are the main responsibilities of your position? |
| Work life balance | How do you balance your work and personal life? Describe a typical day in your life. |
