# Supplementary material for: Understanding key drivers of job satisfaction in primary health care organizations in Kazakhstan: a Herzberg's two-factor theory-based qualitative study
Source: Front Health Serv. 2026 Aug 11;6:1900594. doi: 10.3389/frhs.2026.1900594 (PMC13503204; doi:10.3389/frhs.2026.1900594)
Supplement: Supplementary file 2 [file Table2.docx]

**Table S2. Codebook for qualitative research**

**Research questions:**

1. What are the key drivers of job satisfaction and dissatisfaction among healthcare professionals?
2. What is the effectiveness of social intervention strategies in improving job satisfaction and job performance of healthcare professionals in polyclinic?

Descriptors:

1. Job satisfaction
2. Workload
3. Career growth
4. Recognition
5. Work environment
6. Salary and Benefits
7. Relationship with colleagues
8. Work-life balance

**Table 4.Codebook for qualitative research**

| **Code label** | **Definition** | **Quotes** |  |
| --- | --- | --- | --- |
| Neutral Job Satisfaction | Interviewee says that the work environment in this organization is neutral. | “Overall, the work is not perceived as negative.” P1 |  |
| Autonomy |  |  |  |
| Job Restrictions  Limited Autonomy | Interviewee says that they have a lot of rules and restrictions that they must follow, and being coordinated with the administration creates the limitations in being autonomy. | “In our work there are rules and restrictions that we must follow.” P1  “We must coordinate them with management, justify them, and so on.” P10 |  |
| Comparative workload | Interviewee says that one type of patient (population) creates less workload than having a wide range of patients. | “Of course, the work of a pediatrician is probably a little easier than, for example, that of a general practitioner, because our contingent is only children.” P1 |  |
| Relationships |  |  |  |
| Positive work environment | Interviewee says that the work environment in this organization is positive for her because of the friendly team. | “I really like the clinic because we have a friendly team here.”  P2 |  |
| Supportive colleagues | Interviewees say that  the friendly team makes the work environment positive for them.   Interviewee says that one of the positive factors in the workplace is supportive colleagues. | “I really like the clinic because we have a friendly team here.” P2  “We have a warm and friendly atmosphere, everyone helps each other.” P3 “It's good if there is an adequate nurse who quickly weights and measures the children.”P1  "I enjoy working here because my colleagues are supportive."P5 "We help each other during difficult shifts, which makes things easier."P5 |  |
| Administration support | Interviewee says that the administration creates a warm and cozy atmosphere. | “Yes, there is this coziness and warmth from the management.”P2  “Of course, sometimes there are situations when employees, including myself, may be late due to personal circumstances. We have loyal management that is understanding of such situations.”P3 |  |
| Patient complains | Interviewee says that there are challenges with patients. | “...of course, it is difficult with patients.” P2 "The program often doesn't work, and that causes patient dissatisfaction." P3 |  |
| Positive colleague relationships | Interviewee says that good communication with team members, having meetings with them outside of the job creates positive job environment. | “We communicate well. We often have meetings with the team, where we relax and spend time together. Now we often celebrate holidays, such as New Year, we have corporate parties.”P2  "Good teamwork helps us handle emergencies smoothly."P5 |  |
| Meetings outside of the job | Interviewee says that there  are events outside of work. | “We often have meetings with the team, where we relax and spend time together.”P4 |  |
| Corporate events | Interviewee says that there are two corporate events that they celebrate. | "We have an established tradition of holding two corporate events a year: on Medical Workers Day and on New Year's.”P6 |  |
| Lack of teamwork | Interviewee says that the lack of teamwork addresses dissatisfaction with the workplace. | “Now I work in a public polyclinic, and I don’t like anything here. Firstly, there is no normal team, they do not have teamwork.”P4 |  |
| Love for the profession |  |  |  |
| Desire for teamwork | Interviewee says that there is a lack of teamwork, and it would be a better place if teamwork was established. | “It seems as if everyone is for themselves. I would like to establish teamwork. Before that, I worked in an organization that was half private, half state, but everything was much more difficult there.”P2 |  |
| Passion for profession | Interviewee says that one of the factors of motion is love for profession. | "Only love for my work and love for my patients motivate me."P2  “Working with children is a very special atmosphere. Even if the child is often sick, you still get positive energy from him. That is why I love my work with children so much, and this largely compensates for the numerous disadvantages of my profession.” P1  “When a patient comes with positive feedback, that is, dynamics. They are better and the treatment helps, this is the best. And it seems to me that many doctors only because of this remain in medicine.”P4 "I always wanted to be a doctor; it’s not just a job for me."P5 |  |
| Intrinsic motivation | Interviewee says that one of the factors of motivation is love for profession. | "Only love for my work and love for my patients motivate me." P2 “First of all, I enjoy working with the patients themselves. I love my profession very much.”P6  “To be honest, the most satisfying thing is when we work directly with patients. Well, without, say, dealing with paperwork. Direct work with patients, communication, helping them, treatment, diagnostics.”P3 |  |
| Time limitation |  |  |  |
| Lack of reflection on motivation | Interviewee says that the factor of  overloaded work does not give a chance to think about motivation. | "There is no time to think about motivation in the clinic.”P7 |  |
| Limited time | Interviewee says that the allocated time (15 minutes) is not enough to make proper examinations of the patient. | “I only had 15 minutes at the clinic, and I didn’t understand what to do: look at the throat, write a chart, or undress the child.” P1  "There's just not enough time for anything."P6 |  |
| Workload |  |  |  |
| Negative attitude to home visits | Interviewee says that  sometimes have to be made in bad weather. | "I hated “home visits”, especially in winter."P1 |  |
| Physical exhaustion | Interviewee says that sometimes due to the workload she got Work fatigue | "I came home after work and couldn't even talk."P3 |  |
| Excessive paperwork | Interviewee says that they have excessive paperwork. | "You work, and then you fill out a bunch of paperwork."P1 "The appointment takes 4 hours, and another 2 hours for paperwork."P7 |  |
| Paperwork for disabled patients | Interviewee says that there are a lot of paperwork | "Especially if there are disabled people on the site who need to undergo examinations every year."P1 |  |
| Disabled patients documentation | Interviewee says that there are a lot of paperwork | “You work, and then you fill out a bunch of paperwork, especially if there are disabled people on the site who need to undergo examinations every year.”P6 |  |
| Work family conflict |  | “My daughter suffered because I was constantly absent. I remember one time I stayed late at work and my daughter cried for two hours non-stop. After that, I decided to quit.”P1 |  |
| Dependence of workload on population |  | “But my colleague, for example, is in a better situation because she has a good site, the EXPO area, and intelligent patients.”P1 |  |
| Extreme work requirements |  | “But I objected and was indignant because I had to go on home visits in 35-degree frost.”P2 |  |
| Experience reduces stress |  | “Now it is less stressful because I've gained experience." P3 |  |
| Negative perception of work |  | “Although I was a young specialist at the time, I asked for a job after the quarantine because I couldn't sit at home. But the work turned out to be very difficult.” P8 |  |
| No work-life balance |  | “All day long, just patients and paperwork, no time for yourself." P1 "We sacrifice our family and personal life, and spend almost all our time at work.” P6 |  |
| Furniture and equipment issues |  | "There are problems with furniture, electrical appliances, and the Internet." P3 |  |
| Dissatisfaction with workplace | Interviewee says that the lack of teamwork addresses dissatisfaction with the workplace. | “Now I work in a public polyclinic, and I don’t like anything here. Firstly, there is no normal team, they do not have teamwork.” P2 |  |
| Not stable internet connections |  | "The program often doesn't work, and that causes patient dissatisfaction." P5 |  |
| Key responsibilities |  | “In general, my duties include managing the area, i.e. various preventive examinations. Since I work with children, these are age-specific preventive examinations, screenings, registering children with certain diagnoses, and referrals for hospitalization. Vaccination and education of the population in my area about the need for vaccination are mandatory.”P1 |  |
| Dependence on protocols |  | “Yes, we can, but this needs to be clearly justified, referring to some protocols for treatment, diagnostics, and so on.”P1 |  |
| Career growth |  |  |  |
| Limited career growth | Interview says that they do not have a wide range of opportunities for career development. | “The prospects for growth within the clinic are probably limited to the position of department head, but personally I am not particularly interested in this.” P1  “Because this position is far from practice. It is mainly management work, work with documentation, etc. I would like to be closer to patients.”P4  "I want to grow in my career, but promotion opportunities are rare."P5 |  |
| Focus on career growth |  | “In my career, I strive more for professionalism as a doctor than for career growth as such. At the moment, this is my priority, because I want to be a practitioner.”P10 |  |
| **Education** |  |  |  |
| Confirmation of specialist certificate | Interviewee says that they have continuus education and trainings. | “This is our main task, and to confirm the specialist certificate we must accumulate a certain number of hours.”P1 |  |
| Mandatory trainings |  | "Advanced training is a must."P1 |  |
| Self initiated trainings |  | “...I take courses that interest me, even if they don't count toward my certification hours.”P5 |  |
| Undermining doctor’s role |  | “In general, of course, you feel your value and importance, but, on the other hand, the current reality is that the role of a doctor is somewhat diminished. We are perceived more as service personnel than as specialists.”P1 |  |
| Customer centric policy |  | “They often adopt the 'customer is always right' tactic, although this is far from always the case.”P1 |  |
| Comparison with other countries |  | “Unfortunately, I think the pay is insufficient, especially when compared to Europe, America, etc. There, they won't call a doctor at 3am for no reason, and their income is, of course, higher.”P1 |  |
| Extended vacation |  | “In general, of course, vacation and health benefits exist. Yes, a certain amount is given out. You can get good money during vacation, that's a fact. Doctors have longer vacations, up to 40-45 days, like teachers.”P1 |  |
| Salary and Benefits |  |  |  |
| Workload Pay Imbalance |  | “I think that doctors here don't earn enough, especially in clinics, district doctors, etc. Their work means being busy almost 24/7.”P1 |  |
| Dissatisfaction with salary Discrepancy in Earnings and Responsibility Workload vs Salary | The direct expression of financial dissatisfaction suggests that salary remains a primary concern for medical workers. The use of *"просто"* implies that the request is not excessive but rather a basic expectation. | "The salary does not match the workload; I have to work for several doctors."P2 “But the salary is, of course, a shame, because now there are a lot of When you earn easy money, and you, being responsible for the lives and patients, earn less than a salesperson in a supermarket?”P4 "Overall, I would just like a higher salary."P4 "The SCP is, yes, a nice bonus, but if we are going to improve something, it would be better to raise wages."P4 |  |
| Lack of social benefits |  | “Doctors have longer vacations, up to 40-45 days, like teachers. But doctors cannot get on the waiting list to get an apartment. Or maybe it is only available to those liable for military service, I don't know.”P1 |  |
| Lack of Transparency in Financial Calculations | The distribution of payments depends on internal management decisions, which creates a feeling of unfairness among employees. | “But this money is distributed in a strange way. The deputy head physician and the head of the department sign some indicators, and each time it comes out differently. We have noticed that employees who have been working for a long time receive a little more. Maybe this is some kind of incentive for them.”P4 “For example, if there is some error, of course, we know why we have such a small QC, but there is no interaction with the  accounting department. There are quality indicators that we submit, but they do not give a complete picture.”P4 |  |
| Management | There is a discrepancy between management's stated openness to questions and its actual response, which discourages proactive problem solving. This creates a culture in which employees are hesitant to seek guidance. | "Management always says, 'If you have questions, so that there won't be problems later, come to us, ask questions.' But when you come to management with questions, they first scold you."P4 |  |
|  | A lak of professional bundaris and respect in workplace communication is highly lit, contributing to a toxic environment. | "Even if it's not your fault. There are complaints, but in general they are almost always unfounded, and this is accompanied by shouting."P2 |  |
| Bureaucratic and Selective Managerial Support |  | "The management, the most they can do is help to get the patient through faster if you say that he is in serious condition and needs urgent care."P7  "They can help because they have connections and can call the management of other large clinics."P5 |  |
