# Supplementary material for: Understanding key drivers of job satisfaction in primary health care organizations in Kazakhstan: a Herzberg's two-factor theory-based qualitative study
Source: Front Health Serv. 2026 Aug 11;6:1900594. doi: 10.3389/frhs.2026.1900594 (PMC13503204; doi:10.3389/frhs.2026.1900594)
Supplement: Supplementary file 3 [file Table3.docx]

**Table S3. Consolidated criteria for reporting qualitative studies (COREQ): 32-item checklist.**

**Study title:** *Understanding Key Drivers of Job Satisfaction in Primary Health Care Organizations in Kazakhstan: A Herzberg Theory-Based Qualitative Study*

**Authors:** Ainara Oraltayeva¹, Claire Chaumont², Lyazzat Kosherbayeva³, Kuanysh A. Yergaliyev¹ (corresponding author: kxy059@mail.harvard.edu)

**Journal:** BMC Human Resources for Health	|	**Setting:** Public polyclinics, Astana, Kazakhstan	|	**Period:** January–March 2025	|	**Ethics ref:** NUSOM IREC 2024Dec#03

| **No.** | **Domain** | **Item** | **Description** | **Response** | **Location in Manuscript** |
| --- | --- | --- | --- | --- | --- |
| **DOMAIN 1: Research team and reflexivity** | | | | | |
| **1** | *Personal characteristics* | **Interviewer/facilitator** | Which author(s) conducted the interview or focus group? | AO (first author, Nazarbayev University School of Medicine, Astana, Kazakhstan). Interviews were conducted individually by the first author. | *Methods – Participants and Sampling; Data Collection;* |
| **2** | *Personal characteristics* | **Credentials** | What were the researcher's credentials, e.g. PhD, MD? | AO (first author, interviewer): graduate student researcher, Nazarbayev University School of Medicine. C (co-author): DrPH, researcher, Harvard T.H. Chan School of Public Health, Boston, USA. LK (co-author): MD, PhD, Health Policy and Management, Asfendiyarov Kazakh National Medical University, Almaty. KY (corresponding author): MD, MPH, DrPH, Nazarbayev University School of Medicine. | *Title Page – Author affiliations and credentials* |
| **3** | *Personal characteristics* | **Occupation** | What was their occupation at the time of the study? | AO: graduate student researcher. KY (supervisor/PI): academic faculty member, MD, MPH, DrPH. CC: co-supervisor, researcher, Harvard T.H. Chan School of Public Health. LK: academic faculty, Asfendiyarov Kazakh National Medical University. | *Title Page – Author affiliations* |
| **4** | *Personal characteristics* | **Gender** | Was the researcher male or female? | Female: AO, CC, LK.  Male: KY | *Title Page (author name);* |
| **5** | *Personal characteristics* | **Experience and training** | What experience or training did the researcher have? | AO: training in qualitative research methods through academic program. Peer debriefing with research supervisor. No prior clinical employment at polyclinics.  CC, LK, KY: experienced researchers, particularly in qualitative research | *Methods – Trustworthiness* |
| **6** | *Relationship with participants* | **Relationship established** | Was a relationship established prior to study commencement? | No prior relationship. Participants were recruited via direct outreach, institutional flyers, and social media. | *Methods – Participants and Sampling* |
| **7** | *Relationship with participants* | **Participant knowledge of the interviewer** | What did participants know about the researcher? e.g. personal goals, reasons for doing the research | Participants were informed of the study purpose (exploring job satisfaction drivers in public polyclinics) during recruitment and at the start of each interview. The researcher's student status was disclosed. | *Methods – Data Collection; Ethical Considerations* |
| **8** | *Relationship with participants* | **Interviewer characteristics** | What characteristics were reported about the interviewer/facilitator? e.g. bias, assumptions, reasons and interests in the research topic | Researcher reflexivity acknowledged in the Limitations section: first author (Ainara Oraltayeva) is a non-clinician student researcher, which may have influenced interpretation of clinical experience narratives. Study supervised by Kuanysh A. Yergaliyev (MD, MPH, DrPH) and co-authored by Claire Chaumont (Harvard T.H. Chan) and Lyazzat Kosherbayeva (Kazakh NMU). | *Methods – Limitations (reflexivity); Title Page* |
| **DOMAIN 2: Study design** | | | | | |
| **9** | *Theoretical framework* | **Methodological orientation and theory** | What methodological orientation was stated to underpin the study? e.g. grounded theory, discourse analysis, ethnography, phenomenology, content analysis | Directed (framework/deductive) content analysis, guided by Herzberg's Two-Factor Theory (1959) as the a priori conceptual framework. | *Methods – Data Analysis; Conceptual Framework* |
| **10** | *Participant selection* | **Sampling** | How were participants selected? e.g. purposive, convenience, consecutive, snowball | Purposive sampling (initial) supplemented by snowball sampling. Selection criterion: licensed physicians employed ≥6 months in public polyclinics. | *Methods – Participants and Sampling* |
| **11** | *Participant selection* | **Method of approach** | How were participants approached? e.g. face-to-face, telephone, mail, email | Direct outreach, institutional flyers, and social media messaging. | *Methods – Participants and Sampling* |
| **12** | *Participant selection* | **Sample size** | How many participants were in the study? | 17 physicians (14 GPs, 1 paediatrician, 1 surgeon, 1 oncologist) across 3 public polyclinics in Astana. | *Methods – Participants and Sampling; Results opening* |
| **13** | *Participant selection* | **Non-participation** | How many people refused to participate or dropped out? Reasons? | Physicians were hesitant to participate, at least 5 people we have approached refused to participate. |  |
| **14** | *Setting* | **Setting of data collection** | Where was the data collected? e.g. home, clinic, workplace | Face-to-face interviews conducted on-site at polyclinics, or via video platform (Teams/Zoom) according to participant preference. | *Methods – Data Collection* |
| **15** | *Setting* | **Presence of non-participants** | Was anyone else present besides the participants and researchers? | No one presented during interviews | *Methods – Data Collection* |
| **16** | *Setting* | **Description of sample** | What are the important characteristics of the sample? e.g. demographic data, date | n=17; 88% female; specialties: 14 GPs, 1 paediatrician, 1 surgeon, 1 oncologist; 3 polyclinics in Astana; interviews Jan–Mar 2025. Table 1 presents full participant characteristics. | *Methods – Participants and Sampling; Results - Table 1* |
| **17** | *Data collection* | **Interview guide** | Were questions, prompts, guides provided by the authors? Was it pilot tested? | Semi-structured interview guide developed based on Herzberg's Two-Factor Theory domains (motivator factors: achievement, recognition, work itself, responsibility, advancement; hygiene factors: salary, interpersonal relations, supervision, working conditions, company policy). | *Methods – Data Collection; additional materials* |
| **18** | *Data collection* | **Repeat interviews** | Were repeat interviews carried out? If yes, how many? | Not conducted. Single interview per participant. |  |
| **19** | *Data collection* | **Audio/visual recording** | Did the research use audio or visual recording? | All interviews were audio-recorded with informed participant consent. | *Methods – Data Collection* |
| **20** | *Data collection* | **Field notes** | Were field notes made during and/or after the interview or focus group? | Field individual notes were taken during the interview | *Methods – Trustworthiness* |
| **21** | *Data collection* | **Duration** | What was the duration of the interviews or focus group? | 40–60 minutes per interview. | *Methods – Data Collection* |
| **22** | *Data collection* | **Data saturation** | Was data saturation discussed? | Yes. Thematic saturation was reported to have been reached at 17 participants. | *Methods – Participants and Sampling* |
| **23** | *Data collection* | **Transcripts returned** | Were transcripts returned to participants for comment and/or correction? | member checking or transcript return was not conducted. |  |
| **DOMAIN 3: Analysis and findings** | | | | | |
| **24** | *Data analysis* | **Number of data coders** | How many data coders coded the data? | Three researchers were reviewed codes. | *Methods – Data Analysis; Trustworthiness* |
| **25** | *Data analysis* | **Description of the coding tree** | Did authors provide a description of the coding tree? | Codebook developed iteratively using NVivo; initial codebook derived from 20% of transcripts and refined through peer debriefing. Full codebook not appended; thematic structure presented in Table 3. | *Methods – Data Analysis; Results – Table 3* |
| **26** | *Data analysis* | **Derivation of themes** | Were themes identified in advance or derived from the data? | Deductive/a priori derivation: themes derived using Herzberg's Two-Factor Theory as the coding framework. Data mapped to pre-specified motivator and hygiene factor categories. | *Methods – Conceptual Framework; Data Analysis* |
| **27** | *Data analysis* | **Software** | What software, if applicable, was used to manage the data? | NVivo 14 qualitative data management software. | *Methods – Data Analysis* |
| **28** | *Data analysis* | **Participant checking** | Did participants provide feedback on the findings? | Member checking was not conducted. |  |
| **29** | *Reporting* | **Quotations presented** | Were participant quotations presented to illustrate the themes/findings? Was each quotation identified? e.g. participant number | Yes. Direct participant quotations presented for all 9 themes. Each quotation identified by participant code [P1–P17] and specialty (e.g., P4, General Practitioner). | *Results – Themes 1–9 (all quotations)* |
| **30** | *Reporting* | **Data and findings consistent** | Was there consistency between the data presented and the findings? | Yes. Each theme is directly supported by illustrative quotations that align with the theme description. Herzberg framework mapping validated in Table 3. | *Results – all themes; Table 3* |
| **31** | *Reporting* | **Clarity of major themes** | Were major themes clearly presented in the findings? | Yes. Nine themes reported across two categories (4 hygiene factors, 5 motivator factors), each with a distinct heading, narrative description, and supporting quotations. Table 3 provides structured overview. | *Results – Themes 1–9; Table 3* |
| **32** | *Reporting* | **Clarity of minor themes** | Is there a description of diverse cases or discussion of minor themes? | Partially. Theme 9 (Diminished Societal Recognition) is identified as straddling hygiene/motivator boundaries. The Role of Social Interventions is reported as an additional finding. Minor variation by specialty or polyclinic not explicitly discussed. | *Results – Theme 9; The Role of Social Interventions* |

*Reference: Tong A, Sainsbury P, Craig J. Consolidated criteria for reporting qualitative research (COREQ): a 32-item checklist for interviews and focus groups. Int J Qual Health Care. 2007;19(6):349–357.*
